# Preparation of Some Eco-friendly Corrosion Inhibitors Having Antibacterial Activity from Sea Food Waste

**DOI:** 10.1007/s11743-012-1395-3

**Published:** 2012-08-24

**Authors:** Mohamed H. M. Hussein, Mohamed F. El-Hady, Hassan A. H. Shehata, Mohammad A. Hegazy, Hassan H. H. Hefni

**Affiliations:** 1 Petrochemicals Department, Egyptian Petroleum Research Institute (EPRI), Cairo, Egypt; 2 Chemistry Department, Faculty of Science, Al-Azhar University, Cairo, Egypt

**Keywords:** Chitosan, Corrosion, Eco-friendly, Antibacterial, Sea food waste

## Abstract

Chitosan is one of the important biopolymers and it is extracted from exoskeletons of crustaceans in sea food waste. It is a suitable eco-friendly carbon steel corrosion inhibitor in acid media; the deacetylation degree of prepared chitosan is more than 85.16 %, and the molecular weight average is 109 kDa. Chitosan was modified to 2-*N*,*N*-diethylbenzene ammonium chloride *N*-oxoethyl chitosan (compound I), and 12-ammonium chloride *N*-oxododecan chitosan (compound II) as soluble water derivatives. The corrosion inhibition efficiency for carbon steel of compound (I) in 1 M HCl at varying temperature is higher than for chitosan and compound (II). However, the antibacterial activity of chitosan for *Enterococcus faecalis*, *Escherichia coli*, *Staphylococcus aureus*, and *Candida albicans* is higher than for its derivatives, and the minimum inhibition concentration and minimum bacterial concentration of chitosan and its derivatives were carried out with the same strain.

## Introduction

Acid solutions are commonly used in the chemical industry to remove mill scales from metallic surfaces. The addition of inhibitors effectively secures the metal against acid attack. And many studies using organic inhibitors have been reported [1–7]. The inhibitor adsorption mode is strictly affected by its structure. Most acid inhibitors are organic compounds containing oxygen, nitrogen and sulfur. These compounds are adsorbed onto the metallic surface blocking the active corrosion sites. Although the most effective and efficient organic inhibitors are compounds that have π bonds, the biological toxicity of these products, especially organic phosphate, is documented especially with regard to their environmental harmful characteristics [8, 9]. From the standpoint of safety, the development of non-toxic and effective inhibitors is considered most important and desirable. Chitosan is derived from polysaccharide chitin which is well known as a low cost, renewable marine polymer coming from the structural components of the shells of crustaceans, such as shrimps, lobsters, and crabs [10]; it is the most plentiful natural polymer next to cellulose. Chitosan is produced at an estimated amount of one billion tons per year [11]. The molecular structure of chitosan is represented by a beta 1–4 linked linear biopolymer consisting of 80 % poly(d-glucosamine) and 20 % poly(*N*-acetyl-d-glucosamine). Chitosan exhibits various biological activities and biomedical applications including excellent biocompatibility, biodegradability, osteoconductivity, antimicrobial properties, a flocculating agent, a drug delivery vehicle, an immobilization and encapsulation agent of toxic heavy metals, and also in cosmetics [12, 13]. It is a linear polybase electrolyte having a highly positive (C) charge density because it includes an amine group [14]. With such a cationic property together with several hydroxyl groups it is consider a good corrosion inhibitor of steel.

Chitosan inhibits the growth of a fairly diverse range of bacteria [15] and thus offers great benefit to a wide variety of applications, ranging from medical applications [16] to agriculture [17]. The exact mechanism of the antimicrobial action of chitosan is still ambiguous, although six main mechanisms, none of which are mutually exclusive, have been proposed [18, 19] as follows: (1) Interactions between the positively charged moieties on the chitosan molecules and those negatively charged ones on the microbial cell outer membranes, lead to changes in the cell membrane structure and permeability. This induces the leakage of proteinaceous and other intracellular constituents and so challenges the biochemical and physiological competency of the bacteria leading to loss of replicative ability and eventual death. (2) Chitosan acts as a chelating agent that selectively binds trace metals and subsequently inhibits the production of toxins and microbial growth. (3) Chitosan activates several defense processes in the host tissue, acts as a water binding agent and inhibits various enzymes. (4) Low molecular weight chitosan penetrates the cytosol of the microorganisms and, through the binding of chitosan with DNA, results in the interference with the synthesis of mRNA and proteins. (5) Chitosan on the surface of the cell can form an impermeable polymeric layer which alters the cell permeability and prevents nutrients from entering the cell. (6) Finally, since chitosan can adsorb the electronegative substances in the cell and flocculate them, it disturbs the physiological activities of the microorganism leading to their death.

The aim of this study is to investigate the inhibition efficiency of chitosan and its derivatives on the carbon steel surface in 1 M HCl solution, using weight loss measurements, and antibacterial activity measurement for different strain.

## Materials and Methods

The shrimp shell came as sea food waste from Egyptian shops. Sodium hydroxide, hydrochloric acid, acetone, monochloro acetic acid, *N*,*N* diethyl aniline and 12-aminododecanoic acid were from Sigma Aldrich.

### Extraction of Chitosan

The shrimp shells were deproteinized, demineralized and subsequently decolorized as described in the literature [20–22]. The removal of acetyl groups from the prepared chitin was achieved by mixing with NaOH (50 %) with stirring for 2 h at 115 °C. The resulting chitosan was washed until neutrality with running tap water, rinsed with distilled water, filtered, and then dried at 60 °C for 24 h.

### Preparation of Chitosan Derivatives

**Scheme 1:**
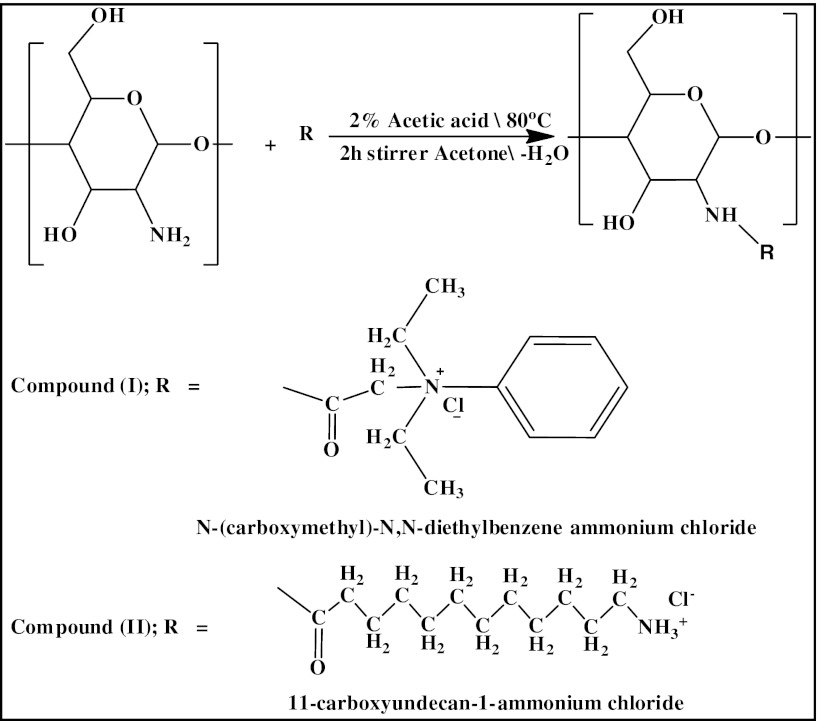
Preparation of chitosan derivatives

The two derivatives of chitosan were prepared as shown in Scheme 1.

#### Preparation of 2-*N*,*N*-Diethylbenzene Ammonium Chloride *N*-Oxoethyl Chitosan (Compound I) in Two Steps

Quaternization of *N*,*N* diethyl aniline by mono chloro acetic acid to produce *N*-(carboxymethyl)-*N*,*N*-diethyl benzene ammonium chloride.

A mixture of *N*,*N* diethyl aniline (0.1 mol; 14.9 g), chloro acetic acid (0.1 mol; 9.4 g) and 100 ml acetone were refluxed for 72 h at 60 °C until a dark green solution was obtained. The product was then cooled, filtered and dried by vacuum distillation.

The structure of this compound was confirmed by FT-IR and ^1^H NMR.

#### Preparation of 2-*N*,*N*-Diethyl Benzene Ammonium Chloride *N*-Oxoethyl Chitosan

A chitosan sample (2 g) was dissolved in an aqueous solution of 2 % V/V acetic acid by vigorously stirring to obtain a solution with a concentration of 2 %, filtered through polyester cloth to remove residues of insoluble particles [23]; the desired amount of *N*-(carboxymethyl)-*N*,*N*-diethylbenzene ammonium chloride (mol/mol amine group of chitosan) was added to the chitosan solution. After agitating for 2 h at 80 °C the 2-*N*,*N*-diethylbenzene ammonium chloride *N*-oxoethyl chitosan was precipitated by acetone, filtered, washed several times with acetone, and dried in a desiccator for 24 h.

#### Preparation of 12-Ammonium Chloride *N*-oxododecan Chitosan (Compound II)

The desired amount of 12-aminododecanoic acid (mol/mol amine group of chitosan) was dissolved in 60 ml of 0.1 M HCl and added to the 2 % chitosan solution with stirring for 2 h at 80 °C, the 12-ammonium chloride *N*-oxododecan chitosan was precipitated by acetone, filtered, washed several times with acetone, and dried in a desiccator for 24 h.

### Characterization of the Prepared Compounds

FT-IR measurement was carried out using a Shimadzu FTIR-4200 spectrometer with a wave number range of 400–4,200 cm^−1^ and resolution 100 cm^−1^.

The elemental analyses were carried out for all prepared compound using a CHNS/O analyzer (Perkin-Elmer, USA), and lasted in Table 1.

**Table 1:** The elemental analysis of chitosan and its derivatives

Compounds	C %	H %	N %	Cl %
Chitosan				
Calculated	44.02	7.10	7.96	–
Found	43.92	7.25	8.11	–
Compound (I)				
Calculated	35.42	3.80	3.55	5.15
Found	35.62	3.82	5.62	5.32
Compound (II)				
Calculated	32.10	3.98	5.01	12.25
Found	32.52	4.40	5.33	12.78

The molecular weight determinations were carried out by gel permeation chromatography (GPC) using a Supremamax 3000 column (Polymer Standard Service, Mainz, Germany) with 2 % CH_3_COOH/0.2 M buffer (CH_3_COONa) as an eluent (1 ml/min). The standard pullulans (*M*_w_ of 11,800, 47,300, 112,000, and 780,000) were used for calibration.

Determination of degree of deacetylation of chitosan (DD) by infrared spectroscopy (FT-IR) and elemental analysis.

### Weight Loss Measurements

The carbon steel specimens have a composition of (wt%): 0.21 C, 0.035 Si, 0.25 Mn, 0.082 P, with the remainder being Fe. The carbon steel sheets of 2.5 cm × 2.0 cm × 0.6 cm were abraded with emery papers (grades 320, 500, 800 and 1200) and then washed with distilled water and acetone. After weighing accurately, the specimens were immersed in 250-mL beakers containing 200 mL of 1 M hydrochloric acid in the absence and in the presence of 10^−8^, 10^−7^, 10^−6^, 10^−5^ and 10^−4^ molar units (monomer) of the inhibitors at 25 °C. After immersion time intervals of 18 h, the specimens were taken out, washed, dried, and weighed accurately. The tests were repeated at 35, 45 and 55 °C. The corrosion rate (*C*_R_) and the inhibition efficiency (η %) were calculated using Eqs. (1–2) [24]:

1 $$ C_{\text{R}} = \frac{W}{St} $$

2 $$ \eta \% = \frac{{C_{\text{R}} - C_{\text{R(inh)}} }}{{C_{\text{R}} }} \times 100 $$

where *W* is the average weight loss of three parallel carbon steel sheets (one specimen in each beaker), *S* is the total area of the steel specimen, and *t* is immersion time, *C*_R_ and *C*_R(inh)_ are the corrosion rates obtained in the absence and the presence of inhibitors, respectively.

The degree of surface coverage θ for different concentrations of the inhibitor in acidic media was evaluated from the weight loss using the equation:

3 $$ \theta = 1 - \frac{{C_{\text{R(inh)}} }}{{C_{\text{R}} }} $$

### Antibacterial Activity of Chitosan and Its Derivatives

#### Bacterial Strain and Inoculum Preparation

Overnight cultures of the following micro-organisms were used throughout the study: *Enterococcus faecalis* as Gram-negative bacteria, *Escherichia coli* as Gram-positive bacteria*, Staphylococcus aureus* as antibiotic resistant bacteria *and Candida albicans* as yeast. Long term maintenance of the microbial strains was at −20 °C using glycerol and short term maintenance was on nutrient agar plates and Sabarouds dextrose agar at 4 °C.

#### Preparation of Solutions

Stock solutions of final concentrations of 2 % chitosan solution, 2 % compound (I) and 2 % of compound (II) were prepared and sterilized.

#### Formation of Clear Zone

Preliminary screening of antimicrobial activity of compounds under investigation was determined by the agar diffusion method, using the cub plate method (II). The petri dishes were incubated at 35 °C for 24 h, except for *C. albicans* cases which were incubated at 27 °C for 48 h. The inhibition zones were measured and recorded as a mean diameter of 3 mm.

#### Minimum Inhibitory Concentration (MIC) Determination

The lowest concentration of antimicrobial activity that inhibits the growth of microorganism being tested as detected by lack of visual turbidity, is known as the minimum inhibitory concentration (MIC). The MIC values of chitosan and its derivatives were determined in duplicate using the twofold broth micro dilution method according to the Clinical and Laboratory Standards Institute (CLSI) [25].

#### Minimum Bacterial Concentration (MBC) Determination

After MIC testing, the microtiter plates setup for the MIC determination was used to determine the MBC. For each sample, 100 μl was transferred and added to 100 μl of saline or 1 % CaCl_2_ solution to neutralize chitosan and its derivatives by dilution and chelation respectively. The entire volume was spread over nutrient agar plate. The MBC point is defined as the lowest concentration showing no growth after incubation.

## Results and Discussion

### Chemical Structures Conformation of Prepared Compounds

#### FTIR Data

FTIR analysis is proposed in many references as a possible way to investigate the interaction between substances [26–28].

**Fig. 1:**
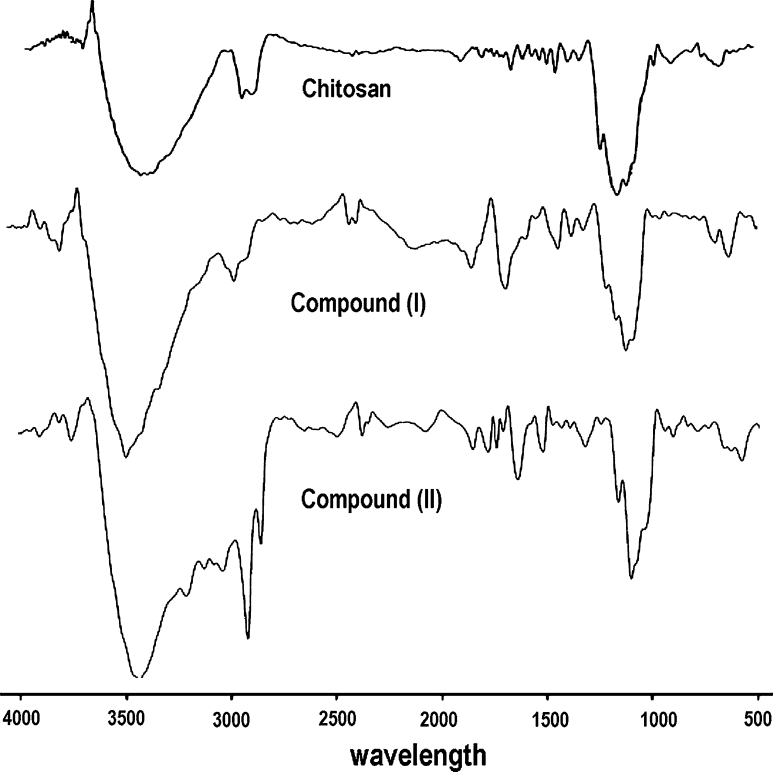
FT-IR spectra of chitosan and its derivatives

In this study, dried chitosan was analyzed by FTIR to observe the possible interaction of the functional groups of both molecules. Figure 1 shows the main bands of chitosan and its derivatives. Chitosan exhibits main characteristic bands of carbonyl (RC=O) and amine group (–NH_2_) at 1,654 and 1,540 cm^−1^, respectively [29, 30]. The broad band due to the stretching vibration of –NH_2_ and –OH group can be observed at 3,400–3,500 cm^−1^ [31, 32]. The bands at 1,000–1,200 cm^−1^ are attributed to the glucosidic ring of chitosan [33]. In the FTIR spectra of compound (I) the same band as found in chitosan, except that the band at 3,450 cm^−1^ is sharp and the band at 1,650 cm^−1^ more intensive. The (NH_2_) group band was shifted to 3,230 cm^−1^ due to the interaction of the amino group. The quaternary ammonium group was observed at 2,615 cm^−1^. In the case of compound (II) the high intensity band is found at 2,920 and 2,880 cm^−1^ related to (CH_2_) of the hydrocarbon chain; the carbonyl group binding amide is observed at 1,650 cm^−1^, and the other bands are the same in chitosan and compound (I).

#### Determination of Degree of Deacetylation (DD)

The degree of deacetylation (DD) of prepared chitosan can be calculated by:

##### FTIR Spectroscopy

By applying [34, 35] the following Eq. (4) the DD equal to 88.15 %.

4 $$ {\text{DD}} = 97.67 - \left[ {26.486\left( {\frac{{A_{1,655} }}{{A_{3,450} }}} \right)} \right] $$

where *A*_1,655_ and *A*_3,450_ is the tow absorbance bands at 1,655 and 3,450 cm^−1^ which related to amide and amine groups respectively.

##### Elemental Analysis

The DD equal to 82.17 % according to Eq. (5)

5 $$ {\text{DD}} = \left( {\frac{{6.857 - {\text{C}}/{\text{N}}}}{1.743}} \right) \times 100 $$

where C/N is the ratio carbon/nitrogen [36] as determined by elemental analysis.

The average degree of deacetylation (DD) [36] of prepared chitosan can be calculated from Eq. (6).

6 $$ \overline{\text{DD}} \% = \frac{{{\text{DD}}_{\text{IR}} + {\text{DD}}_{\text{CHN}} }}{2} = 85.16\,\% $$

##### GPC Data

From the GPC data, we found that the molecular weights of chitosan, compound (I) and compound (II) are 109.050, 118.81, and 137.26 kDa respectively. The increase in molecular weight of these compounds over that of chitosan indicated the formation of new products.

### Corrosion Results

#### Effect of Temperature

From corrosion rate values which listed in Table 2, we found that the corrosion rates decrease with increasing concentration of inhibitors, and increased by increasing the temperature, as a result of decreasing the apparent activation energy (*E*_a_) of the charge transfer reaction.

**Table 2:** Weight loss data of carbon steel corrosion in 1 M HCl in absence and presence of different concentrations of the prepared inhibitors at different temperatures

Compounds	Conc. of inhi. (M)	298 K			308 K			318 K			328 K		
		*C*_R_ (g cm^−2^ h^−1^)	θ	η %	*C*_R_ (g cm^−2^ h^−1^)	θ	η %	*C*_R_ (g cm^−2^ h^−1^)	θ	η %	*C*_R_ (g cm^−2^ h^−1^)	θ	η %
Blank	0.00	0.35			0.51			0.93			1.52		
Chitosan	10^−8^	0.21	0.40	40.15	0.33	0.35	34.91	0.68	0.27	26.61	1.27	0.16	16.41
	10^−7^	0.17	0.50	50.32	0.28	0.44	44.44	0.60	0.27	26.61	1.13	0.25	25.39
	10^−6^	0.13	0.64	63.57	0.21	0.58	58.22	0.46	0.35	34.91	1.04	0.31	31.49
	10^−5^	0.04	0.88	88.50	0.10	0.80	80.12	0.27	0.73	73.66	0.53	0.65	65.08
	10^−4^	0.05	0.86	85.52	0.10	0.80	79.91	0.30	0.71	70.91	0.60	0.60	60.41
Compound (I)	10^−8^	0.20	0.43	43.45	0.32	0.37	36.66	0.68	0.26	26.16	1.23	0.19	19.15
	10^−7^	0.16	0.55	55.00	0.28	0.45	45.16	0.56	0.40	39.95	1.06	0.30	30.28
	10^−6^	0.10	0.71	71.31	0.15	0.70	70.38	0.39	0.58	57.96	0.80	0.48	47.51
	10^−5^	0.05	0.86	86.39	0.06	0.87	87.91	0.16	0.83	83.10	0.50	0.67	67.04
	10^−4^	0.06	0.85	84.83	0.07	0.88	88.50	0.19	0.79	79.39	0.54	0.64	64.19
Compound (II)	10^−8^	0.24	0.31	31.44	0.36	0.29	28.97	0.74	0.20	20.24	1.30	0.14	14.24
	10^−7^	0.22	0.38	38.31	0.33	0.35	35.12	0.69	0.26	25.68	1.18	0.22	21.89
	10^−6^	0.15	0.57	56.97	0.20	0.61	60.75	0.50	0.46	45.92	1.05	0.31	30.81
	10^−5^	0.08	0.77	77.18	0.11	0.79	79.03	0.29	0.69	69.14	0.57	0.62	62.19
	10^−4^	0.09	0.75	75.48	0.12	0.77	76.78	0.29	0.69	68.54	0.61	0.59	59.45

The increase in temperature will enhance the rate of H^+^ diffusion to the metal surface as well as ionic mobility. At lower temperatures, the adsorbed hydrogen atoms block the cathodic area, while the increase in the solution temperature causes desorption of hydrogen. Such hydrogen desorption leads to an increase in the cathodic area and consequently increases the corrosion rate. This behavior is repeated for all compounds. These results showed that the prepared compounds act as efficient inhibitors at lower temperatures rather than at high temperatures.

This behavior was the same for all the prepared inhibitor compounds.

#### Inhibition Efficiency of Inhibitors

However, the data in Table 2 describe that the inhibition efficiency increases with increasing concentration of prepared inhibitors, and decreases with increasing temperature, while in the case of compounds (I) and (II), the values of inhibition efficiencies are higher at 308 K than at 298 K, a trend that could be due to the higher solubility of these compounds at 308 K.

#### Adsorption Isotherms

The prepared compounds inhibit the corrosion process by adsorption on the metal surface. As it is known, the adsorption of inhibitor (*I*_ads_) is always a displacement reaction involving removal of “*x*” number of the absorbed water molecules from the metal surface, according to the Eq. (7):

7 $$ {\text{I}}_{\text{aq}} + x{\text{H}}_{2} {\text{O}}_{\text{ads}} \to I_{\text{ads}} + \, x{\text{H}}_{2} {\text{O}}_{\text{aq}} $$

The adsorption depends on the structure of the inhibitor, the type of the metal and the nature of its surface, pH of the corrosion medium, the temperature, and the electrochemical potential of the metal–solution interface.

The mathematical relationship for the adsorption isotherms suggested that the experimental data of the present work fit the Langmuir model [37, 38] in the Eq. (8).

8 $$ \frac{C}{\theta } = \frac{1}{{K_{\text{ads}} }} + C $$

where “*K*_ads_” is the equilibrium constant of the adsorption reaction, and *C* is the concentration of inhibitors in the solution bulk.

**Fig. 2:**
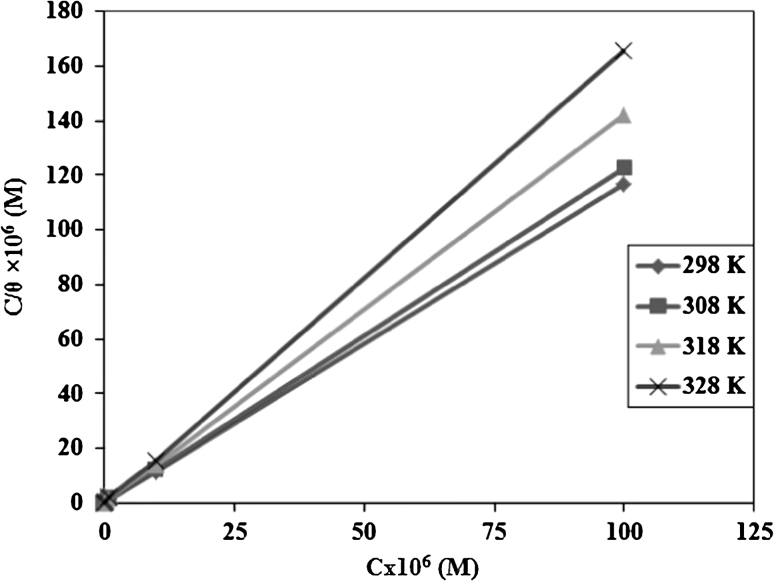
Langmuir’s adsorption plots for carbon steel in 1 M HCl containing different concentrations of chitosan at various temperatures

Figure 2 show the plotting *C*/θ versus *C* which yielded a straight line with a correlation coefficient (*r* ^2^) higher than 0.9999 and a slope close to 1. This indicates that the adsorption of these inhibitors can be fitted to a Langmuir adsorption isotherm. The strong correlation of the Langmuir adsorption isotherm may confirm the validity of this approach. The equilibrium constant (*K*_ads_) for the adsorption–desorption process of these compounds can be calculated from the reciprocal of the intercept. The adsorptive equilibrium constant (*K*_ads_) values are listed in Table 3. It is clear that, the large values indicate that each inhibitor unit occupies more than one adsorption site on the steel surface, and that there is a strong adsorption of the prepared inhibitors on the surface of carbon steel in 1 M HCl [39].

**Table 3:** Standard thermodynamic parameters of adsorption on carbon steel surface in 1 M HCl containing different concentrations of the prepared compounds

Compounds	Temperature (K)	*K*_ads_ (×10^−6^ M^−1^)	∆*G*_ads_^o^ kJ mol^−1^	∆*H*_ads_^o^ kJ mol^−1^	∆*S*_ads_^o^ J mol^−1^ K^−1^
Chitosan	298	26.07	−42.29	17.18	199.56
	308	34.99	−44.46		200.13
	318	41.38	−46.84		201.31
	328	49.08	−48.09		199.66
Compound (I)	298	183.52	−47.12	−23.41	79.57
	308	126.95	−47.76		79.06
	318	93.84	−48.51		78.94
	328	77.93	−49.53		79.64
Compound (II)	298	9.73	−39.85	−10.77	97.58
	308	38.90	−44.73		110.27
	318	21.74	−44.65		106.53
	328	7.19	−43.04		98.38

### The Thermodynamic Parameters of Adsorption Processes

The free energy of adsorption ∆*G*_ads_^o^ was calculated using the following Eq. (9) [40]:

9 $$ \Updelta G^{\text{o}} = - {\text{RT}}\ln K_{ads} $$

where *R* is the gas constant (8.314 J mol^−1^ K^−1^), *T* is the absolute temperature.

Values of ∆*G*_ads_^o^ are listed in Table 3. The negative values of ∆*G*_ads_^o^ are usually characteristic of spontaneity of the adsorption processes [41]. Generally, values of ∆G_ads_^o^ around −40 kJ mol^−1^ or more involve charge sharing or transfer from the inhibitor molecules to the metal surface to form a coordinate type of bond (chemisorption). Whereas, the negative values of −20 kJ mol^−1^ or lower are consistent with the electrostatic interaction between the charged molecules and the charged metal (physisorption) [42, 43]. Calculated ∆G_ads_^o^ values indicated that the adsorption mechanism of the prepared compounds on carbon steel in 1 M HCl solution is a chemical adsorption [44].

From the plotting of ln *K*_ads_ versus 1/*T*, the heat of adsorption (*Q*_ads_), which is obtained from the slopes of the linear portion of the curve, is equal to −*Q*_ads_/*R*. The value of *Q*_ads_ is equal to enthalpy of adsorption ∆*H*_ads_^o^ with good approximation, because pressure is constant [45], and the equation modified to the following:

10 $$ \ln \, K_{\text{ads}} = ( - \Updelta H^{\text{o}} /{\text{RT}}) + {\text{constant}} $$

The ∆*H*_ads_^o^ values were equal to 17.18, −23.41 and −10.77 kJ mol^−1^ for chitosan, compound (I) and compound (II), respectively. The positive value of ∆*H*_ads_^o^ indicated that the adsorption of chitosan on the carbon steel surface is endothermic, while the negative values indicated that the adsorption of compounds (I) and (II) is exothermic.

Entropy of inhibitor adsorption ∆*S*_ads_^o^ can be calculated using the following Eq. (11) [46]:

11 $$ \Updelta S_{\text{ads}}^{\text{o}} = \left( {\Updelta H_{\text{ads}}^{\text{o}} - \Updelta G_{\text{ads}}^{\text{o}} } \right)/T $$

Also, the positive values of ∆*S*_ads_^o^ indicate the random (disorder) in the layer adsorbed on the solid surface, and attributed to the increase disordering the adsorptions of an inhibitor molecule by desorption of more water molecules.

### Activation Energy

**Fig. 3:**
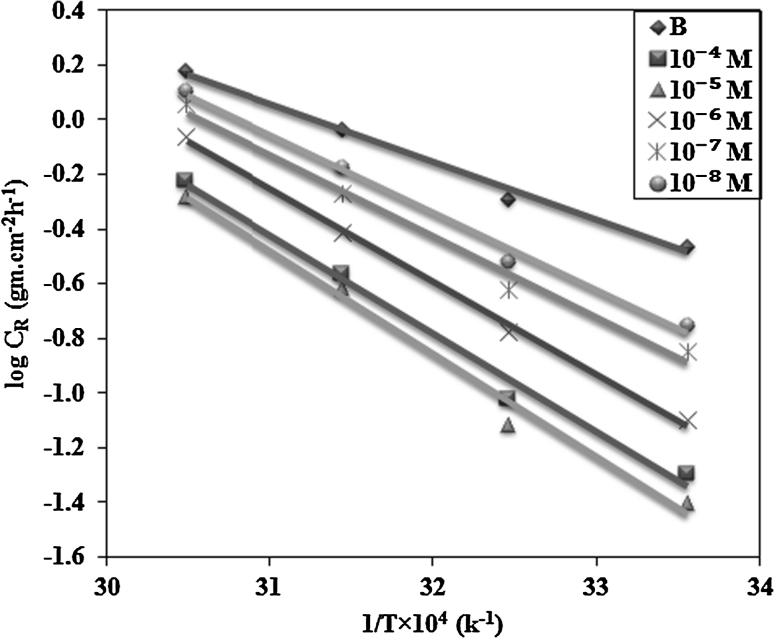
Arrhenius plots of log (*k*/*T*) versus 1/*T* for carbon steel in 1 M HCl solution without and with different concentrations of prepared chitosan

The apparent activation energy, *E*_a_, of the corrosion reaction was determined using Arrhenius plots. The Arrhenius equation can be written as the following Eq. (12) [47, 48]:

12 $$ C_{\text{R}} = \, A \, \exp ( - E_{\text{a}} /{\text{RT}}) $$

where *C*_R_ is the corrosion rate, *E*_a_ is the apparent activation energy of the corrosion reaction, *R* is the gas constant, *T* is the absolute temperature and *A* is the Arrhenius pre-exponential factor. The apparent activation energy of the corrosion reaction in the presence and absence of the inhibitors could be determined by plotting log *C*_R_ against 1/*T*, which gives a straight line with a slope permitting the determination of *E*_a_. Figure 3 shows these plots in the absence and presence of different concentrations of inhibitors. The calculated values of the apparent activation corrosion energies in the absence and presence of inhibitors are listed in Table 4. The higher activation energy values in the presence of inhibitors support the results obtained from the weight loss and indicate the physisorption of the inhibitors.

**Table 4:** Values of activation energy (*E*_a_) for carbon steel in 1 M HCl in absence and presence of different concentrations of the prepared compounds

Conc. of inhibitors (M)	*E*_a_ (kJ mol^−1^)		
	Chitosan	Compound (I)	Compound (II)
0.00	110.59	110.59	110.59
10^−8^	135.43	137.82	128.01
10^−7^	141.15	141.83	129.32
10^−6^	156.48	158.31	148.84
10^−5^	193.50	175.80	152.36
10^−4^	188.74	174.73	150.39

### Inhibition Mechanism

**Scheme 2:**
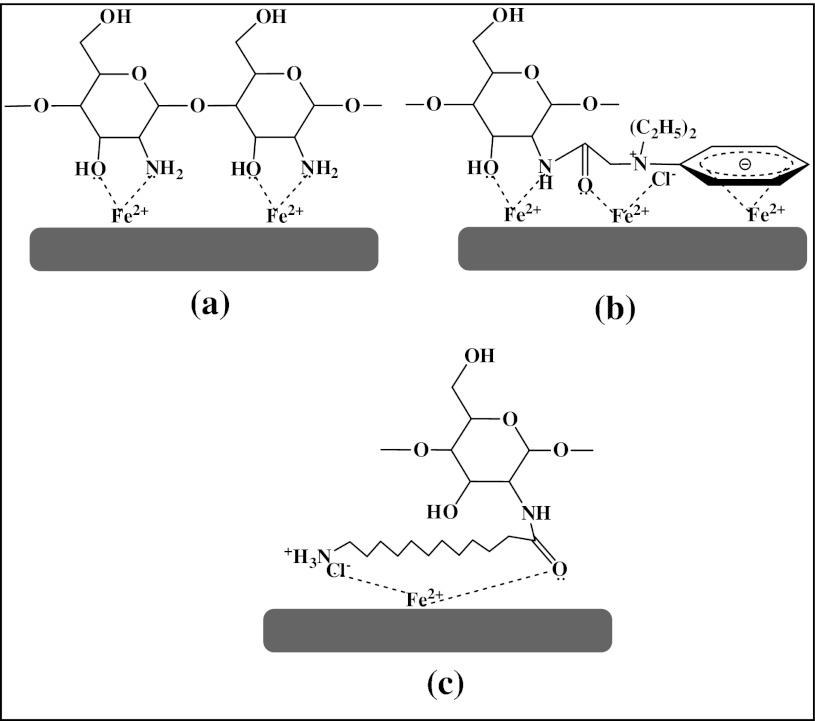
The interaction between steel and inhibitor molecules

The inhibition of the corrosion reaction depends on the better adsorption of inhibitor molecules on the metal surface. Increasing the inhibitor concentration increases the number of the adsorbed molecules which consequently increases the protection of the metal against corrosion. The effectiveness of a compound as a corrosion inhibitor depends on the structure of the inhibitor's compound [49]. The presence of overlapping of intra-hydrogen bonding between the new substitution groups and the chain of bio-polymer plays a major role in increasing η (%) of the inhibitors. Inhibiting the corrosion process in the acidic solutions by the synthesized inhibitors can be explained on the basis of molecular adsorption. It is apparent from the molecular structures that these compounds are able to adsorb on the metal surface through π-electrons of aromatic ring, lone pairs of electrons of N- and O-atoms, and the protonated imine groups (–N=C–) [50]. The investigated compounds in this study exhibit a good performance as corrosion inhibitors. Table 1 showed that compound (I) is more efficient than other compounds. This may be attributed to the orientation of substituted groups and the degree of overlapping of intra-hydrogen bonding within the same molecule, as seen in Scheme 2b which shows the geometrical structure of compound (I) having several active groups such as C=O, (N^+^), OH and NH_2_ groups and π-electrons of aromatic ring for each unit, which facilitate the flat orientation on the surface. In the case of compound (II), the alkyl chain in the substitution groups increased the intra-hydrogen bonding and prevented the mentioned flat orientation on the surface causing less adsorption, and thereby a lower inhibition efficiency. In chitosan alone, no substitution group and a few intra-hydrogen bonding, and the flat orientation on the surface were caring out through OH and NH_2_ groups to result in moderate η (%), as shown Scheme 2a, c.

### Antimicrobial Activity Measurements of Chitosan and Its Derivatives

The inhibition zones of chitosan and its derivatives for *E. faecalis*, *E. coli*, *S. aureus*, and *C. albicans* are tabulated in Table 5 which indicate a better antimicrobial activity of chitosan [51] than its derivatives. This is probably due to the larger number of positive charges encountered in electrostatic interactions between the positively charge of ammonium group of chitosan macromolecule and the negatively charged bacterial cell wall which led to the leakage of proteinaceous and other intracellular constituents. In the chitosan derivatives [52], a lower hydrophilic/hydrophobic balance of charge density leads to a weaker antimicrobial activity.

**Table 5:** The inhibition zones of chitosan and its derivatives for different organisms

Samples	*E. faecalis*	*E. coli*	*S. aureus,*	*C. albicans*
Chitosan	+	++	+++	+
Compound (I)	–	–	+	–
Compound (II)	–	–	–	–

− No zone inhibition, + 1.5:2 cm zone inhibition, ++ 2.5:3.5 cm zone inhibition, +++ 3.9:4.5 cm zone inhibition

### (MIC) and (MBC) Results of Chitosan

From the MIC and MBC results which are listed in Table 6, it is obvious that chitosan has a greater antimicrobial activity for *E. coli* and *S. aureus*, due to the cell wall surface characteristics [53], to be attributed to differences in the hydrophilicity and negative charge distributed on the surface of cell wall [54].

**Table 6:** The results of MIC and MBC of chitosan against different organisms

Microbial strains	MIC mg ml^−1^	MBC mg ml^−1^
*E. faecalis*	3.25 ± 0.0	9.40 ± 1.1
*E. coli*	2.03 ± 0.5	4.80 ± 1.1
*S. aureus*	1.20 ± 0.2	4.90 ± 1.0
*C. albicans*	2.65 ± 0.0	5.28 ± 0.0

## Conclusion

The chitosan and its derivatives were prepared from shrimp shell waste and identified by FTIR, elemental analysis and GPC.

The use of chitosan and its derivatives as corrosion inhibitors for carbon steel in acidic media led to an inhibition efficiency of about 88 % at lower concentration through adsorption processes of the chemical and physical types.

Compound (I) exhibited a higher inhibition efficiency than other compounds, due to a more active side group adsorbed on steel surface. On the other hand, the long chain in compound (II) was coiled on chitosan units and caused a partial steric hindrance and a lower inhibition efficiency.

The antimicrobial activity of chitosan was better than its derivatives due to the stability of charge density in the chitosan chain.

## References

[1] Negm NA, Zaki MF, Salem MAI (2009). Synthesis and evaluation of 4-diethyl amino benzaldehyde Schiff base cationic amphiphiles as corrosion inhibitors for carbon steel in different acidic media. J Surfact Deterg.

[2] Negm NA, Morsy SMI, Said MM (2005). Corrosion inhibition of some novel hydrazone derivatives. J Surfact Deterg.

[3] Negm NA, Morsy MI (2005). Corrosion inhibition of triethanolammonium bromide mono- and dibenzoate as cationic inhibitors in an acidic medium. J Surfact Deterg.

[4] Negm NA, El Farargy AF, Al Sabagh AM, Abdelrahman NR (2011). New Schiff base cationic surfactants: surface and thermodynamic properties and applicability in bacterial growth and metal corrosion prevention. J Surfact Deterg.

[5] Rengamani S, Muralidharan S, Kulamdainathan MA, Venkatakrishna Iyer S (1994). Inhibiting and accelerating effects of aminophenols on the corrosion and permeation of hydrogen through mild steel in acidic solutions. J Appl Electrochem.

[6] Ajmal M, Mideen AS, Quraishi MA (1994) 2-Hydrazino-6-methyl-benzothiazole as an effective inhibitor for the corrosion of mild steel in acidic solutions. Corros Sci 36:79–84

[7] El-Sayed A (1997). Phenothiazine as inhibitor of the corrosion of cadmium in acidic solutions. J Appl Electrochem.

[8] Sinko J (2001). Challenges of chromate inhibitor pigments replacement in organic coatings. Prog Org Coat.

[9] Manahan SE (1996). Environmental chemistry.

[10] Hiano S, Inui H, Kosaki H, Uno Y, Toda T, Gebelein CG, Carraher CE (1994). Chitin and chitosan: ecologically bioactive polymer. Biotechnology and bioactive polymers.

[11] Sugama T, Cook M (2000). Poly(itaconic acid)-modified chitosan coatings for mitigating corrosion of aluminum substrates. Prog Org Coat.

[12] Bumgardner JD, Wiser R, Gerard PD, Bergin P, Chestnutt B, Marini M, Ramsey V, Elder SH, Gilbert JA (2003). Chitosan: potential use as a bioactive coating for orthopaedic and craniofacial/dental implants. J Biomater Sci Polym Edn.

[13] Martino AD, Sittinger M, Risbud MV (2005). Chitosan: a versatile biopolymer for orthopaedic tissue-engineering. Biomaterials.

[14] Sandford PA, Steinners A (1991) Biomedical applications of high-purity chitosan. In: Shalaby SW, McCormick CL, Butles GB (eds) Water-soluble polymers, ACS symposium series 467, Washington, DC, p 430

[15] Choi BK, Kim KY, Yoo YJ, Oh SJ, Choi JH, Kim CY (2001). In vitro antimicrobial activity of a chitooligosaccharide mixture against *Actinobacillus actinomycetemcomitans* and *Streptococcus mutans*. Int J Antimicrob Agents.

[16] Alves NM, Mano JF (2008). Chitosan derivatives obtained by chemical modifications for biomedical and environmental applications. Int J Biol Macromol.

[17] Campaniello D, Bevilacqua A, Sinigaglia M, Corbo MR (2008). Chitosan: antimicrobial activity and potential applications for preserving minimally processed strawberries. Food Microbiol.

[18] Raafat D, von Bargen K, Haas A, Sahl HG (2008). Insights into the mode of action of chitosan as an antibacterial compound. Appl Environ Microbiol.

[19] Fernandez-Saiz P, Lagaron JM, Hernandez-Muñoz P, Ocio MJ (2008). Characterization of antimicrobial properties on the growth of *S. aureus* of novel renewable blends of gliadins and chitosan of interest in food packaging and coating applications. Int J Food Microbiol.

[20] Hussein MHM, El-Hady MF, Sayed WM, Hefni H (2012). Preparation of some chitosan heavy metal complexes and study of its properties. Polym Sci Ser A.

[21] Methacanon P, Prasitsilp M, Pothsree T, Pattaraarchachai J (2003). Heterogeneous N-deacetylation of squid chitin in alkaline solution. J Carbohydr Polym.

[22] Yaghobi N, Hormozi F (2010). Multistage deacetylation of chitin: kinetics study. J Carbohydr Polym.

[23] Abd El-Ghaffar MA, Hashem MS (2010). Chitosan and its amino acids condensation adducts as reactive natural polymer supports for cellulase immobilization. Carbohydr Polym.

[24] ASTM G1-72 (1990) Practice for preparing, cleaning and evaluating corrosion test specimens

[25] National Committee for Clinical Laboratory Standards (1981) Development of in vitro susceptibility testing criteria and quality control parameters: approved standard. In: NCCLS M23-A2 NCCLS, Wayne, PA, USA

[26] Otagiri M, Saito H, Shiraishi S, Imai T (1991). Interaction of indomethacin with low molecular weight chitosan, and improvements of some pharmaceutical properties of indomethacin by low molecular weight chitosan’s. Int J Pharm.

[27] Domard A, Zydowicz N, Vachoud L (1997). Formation and characterization of a physical chitin gel. Carbohydr Res.

[28] Liu W, Sun S, Cao Z, Zhang X, Yao K (2005). An investigation on the physicochemical properties of chitosan/DNA polyelectrolyte complexes. Biomaterials.

[29] Arof AK, Osman Z (2003). FTIR studies of chitosan acetate based polymer electrolytes. Electrochim Acta.

[30] Salokhe VM, Rakshit SK, Pranoto Y (2005). Enhancing antimicrobial activity of chitosan films by incorporating garlic oil, potassium sorbate and nisin. Lebensm Wiss Technol.

[31] Wang X, Du Y, Liu H (2004). Preparation, characterization and antimicrobial activity of chitosan–Zn complex. Carbohydr Polym.

[32] Xu Y, Du Y (2003). Effect of molecular structure of chitosan on protein delivery properties of chitosan nanoparticles. J Pharm.

[33] Mincheva R, Manolova N, Sabov R, Kjurkchiev G, Rashkov L (2004). Hydrogels from chitosan crosslinked with poly(ethylene glycol) diacid as bone regeneration materials. e-Polymers.

[34] Khan TA, Peh KK, Ch’ng HS (2002). Reporting degree of deacetylation values of chitosan: the influence of analytical methods. J Pharm Pharmaceut Sci.

[35] Baskar D, Sampath Kumar TS (2009). Effect of deacetylation time on the preparation, properties and swelling behavior of chitosan films. Carbohydr Polym.

[36] Al Sagheer FA, Al-Sughayer MA, Muslima S, Elsabee MZ (2009). Extraction and characterization of chitin and chitosan from marine sources in Arabian Gulf. Carbohydr Polym.

[37] Riggs OL, Nathan CC (1973). Theoretical aspects of corrosion inhibitors and inhibition. Corrosion inhibitors.

[38] Zhao TP, Mu GN (1999). The adsorption and corrosion inhibition of anion surfactants on aluminium surface in hydrochloric acid. Corr Sci.

[39] Cheng S, Chen S, Liu T, Chang X, Yin Y (2007). Carboxymethyl chitosan as an ecofriendly inhibitor for mild steel in 1 M HCl. Mater Lett.

[40] Quartarone G, Battilana M, Bonaldo L, Tortato T (2008). Investigation of the inhibition effect of indole-3-carboxylic acid on the copper corrosion in 0.5 M H_2_SO_4_. Corros Sci.

[41] Hosseini SMA, Azimi A (2009). The inhibition of mild steel corrosion in acidic medium by 1-methyl-3-pyridine-2-yl-thiourea. Corros Sci.

[42] Hegazy MA (2009). A novel Schiff base-based cationic gemini surfactants: synthesis and effect on corrosion inhibition of carbon steel in hydrochloric acid solution. Corros Sci.

[43] Okafor PC, Zheng Y (2009). Synergistic inhibition behaviour of methylbenzyl quaternary imidazoline derivative and iodide ions on mild steel in H_2_SO_4_ solutions. Corros Sci.

[44] Behpour M, Ghoreishi SM, Soltani N, Salavati-Niasari M, Hamadanian M, Gandomi A (2008). Electrochemical and theoretical investigation on the corrosion inhibition of mild steel by thiosalicylaldehyde derivatives in hydrochloric acid solution. Corros Sci.

[45] Tao Z, Zhang S, Li W, Hou B (2009). Corrosion inhibition of mild steel in acidic solution by some oxo-triazole derivatives. Corros Sci.

[46] Ashassi-Sorkhabi H, Shaabani B, Seifzadeh D (2005). Corrosion inhibition of mild steel by some Schiff base compounds in hydrochloric acid. Appl Surf Sci.

[47] Shukla SK, Quraishi MA (2009). 4-Substituted anilinomethylpropionate: new and efficient corrosion inhibitors for mild steel in hydrochloric acid solution. Corros Sci.

[48] Tang L, Mu G, Liu G (2003). The effect of neutral red on the corrosion inhibition of cold rolled steel in 1.0 M hydrochloric acid. Corros Sci.

[49] Li X, Deng S, Fu H (2011). Sodium molybdate as a corrosion inhibitor for aluminium in H_3_PO_4_ solution. Corros Sci.

[50] Negm NA, Zaki MF, Said MM, Morsy SM (2011). Inhibitory action of biodegradable modified vanillin on the corrosion of carbon steel in 1 M HCl. Corros Sci.

[51] Rabea EI, Badawy ME-T, Stevens CV, Smagghe G, Steurbaut W (2003). Chitosan as antimicrobial agent:  applications and mode of action. Biomacromolecules.

[52] Sajomsang W, Tantayanon S, Tangpasuthadol V, Daly WH (2008). Synthesis of methylated chitosan containing aromatic moieties: chemoselectivity and effect on molecular weight. Carbohydr Polym.

[53] Chung Y-C, Su Y-P, Chen C-C, Jia G, Wang H-L, Wu JCG, Lin J-G (2004). Relationship between antibacterial activity of chitosan and surface characteristics of cell wall. Acta Pharmacol Sin.

[54] Kong M, Chen XG, Xing K, Park HJ (2010). Antimicrobial properties of chitosan and mode of action: a state of the art review. Int J Food Microbiol.

